# Risk of Systemic Lupus Erythematosus in Patients With Anti-phospholipid Syndrome: A Population-Based Study

**DOI:** 10.3389/fmed.2021.654791

**Published:** 2021-05-10

**Authors:** Hsin-Hua Chen, Ching-Heng Lin, Wen-Cheng Chao

**Affiliations:** ^1^Department of Medical Research, Taichung Veterans General Hospital, Taichung, Taiwan; ^2^Division of Allergy, Immunology and Rheumatology, Department of Internal Medicine, Taichung Veterans General Hospital, Taichung, Taiwan; ^3^Institute of Biomedical Science and Rong Hsing Research Centre for Translational Medicine, Chung Hsing University, Taichung, Taiwan; ^4^Department of Industrial Engineering and Enterprise Information, Tunghai University, Taichung, Taiwan; ^5^Institute of Public Health and Community Medicine Research Center, National Yang-Ming University, Taipei, Taiwan; ^6^Department of Healthcare Management, National Taipei University of Nursing and Health Sciences, Taipei, Taiwan; ^7^Department of Public Health, College of Medicine, Fu Jen Catholic University, New Taipei City, Taiwan; ^8^Department of Critical Care Medicine, Taichung Veterans General Hospital, Taichung, Taiwan; ^9^Department of Computer Science, Tunghai University, Taichung, Taiwan; ^10^Department of Automatic Control Engineering, Feng Chia University, Taichung, Taiwan

**Keywords:** anti-phospholipid syndrome, systemic lupus erythematosus, autoimmune diseases, risk, propensity matching

## Abstract

**Objective:** To investigate the association between anti-phospholipid syndrome (APS) and the risk of newly diagnosed systemic lupus erythematosus (SLE).

**Methods:** We used 2003–2013 data derived from Taiwan's National Health Insurance Research Database to conduct this nationwide, population-based. We identified AS patients newly diagnosed between 2005 to 2013 as the study group and applied age-sex matched (1:20) and propensity score-matched (PSM) (1:2) non-SLE individuals as controls. The association between APS and risk of incident SLE was determined by calculating hazard ratios (HRs) with 95% confidence intervals (CIs) using Cox proportional hazard regression analysis.

**Results:** We identified 1,245 patients with APS as well as 24,900 age- and sex-matched non-APS controls and 727 APS patients as well as 1,454 PSM non-APS controls. We found that the risk for incident SLE in the APS group was 80.70 times higher than the non-APS group, and the association remained robust after PSM (HR, 28.55; 95% CI, 11.49–70.91). The increased risk for SLE in patients with APS mainly existed within 5 years after the diagnosis of APS. The sensitivity analyses found that the risk for SLE in patients with APS was consistent excluding patients with ITP/AIHA and using distinct definitions of SLE.

**Conclusion:** The present population-based study revealed a robust association between SLE risk and recent APS and highlights the need for vigilance of SLE-associated symptoms in patients who had been diagnosed with APS.

## Introduction

Systemic lupus erythematosus (SLE) can manifest with haematological and vascular abnormalities prior to the diagnosis of SLE, and recent studies including our study have found an increased risk for SLE in patients with haematological abnormalities, including immune thrombocytopenia (ITP) and autoimmune hemolytic anaemia (AIHA) (1, 2). However, clinical evidence with regards to the association between vascular abnormalities and SLE remains sparse.

Anti-phospholipid syndrome (APS), characterised by obstetric morbidities and/or arterial/venous thrombosis, has been highly implicated with SLE. APS and SLE are two closely correlated autoimmune diseases with overlapped clinical and biological characteristics (3–5). Approximately 30% of APS has been reported to be associated with SLE (6, 7). Cervera et al. conducting a 10-year-follow-up study among 1,000 patients with APS in 13 European countries, reported that 36.2% of APS was associated with SLE (7). Furthermore, the anti-phospholipid antibodies were found in ~40% of patients with SLE (6, 8). Additionally, SLE-associated APS was found to be more likely to have thrombocytopenia than patients with primary APS (7, 9). Therefore, APS appears to be associated with the development of SLE, and we hence aim to investigate the association between APS and incident SLE. In the present study, we used a nationwide population-based database and propensity matching to address the strength and time-course of association between APS and the development of SLE.

## Materials and Methods

### Ethics Statement

The Institutional Review Board of Taichung Veterans General Hospital in Taiwan approved the present study (approval number: CE17100B). The requirement for informed consent was waived due to that the data in the present study were anonymised before analyses.

### Data Sources

The data used in this study were derived from the National Health Insurance Database (NHID). The Taiwan National Health Research Institutes (NHRI) collected and maintained the enrollment files and original reimbursement claims data from the National Health Insurance (NHI) administration, and then released them to the NHID. The database includes the stored medical claims from 1997 to 2013 for nearly 99% of the 23.74 million Taiwanese residents. Diagnosis of inpatients and outpatients based on the International Classification of Diseases, Ninth Revision, Clinical Modification (ICD-9-CM), clinical examinations, prescriptions, and medical expenses are recorded in this database, which can be used to study the incidence and correlation of diseases. However, the NHIRD lacked laboratory data and some personal information such as smoking, drinking, body weight, and body length.

### Study Design and Participants

**Figure 1:**
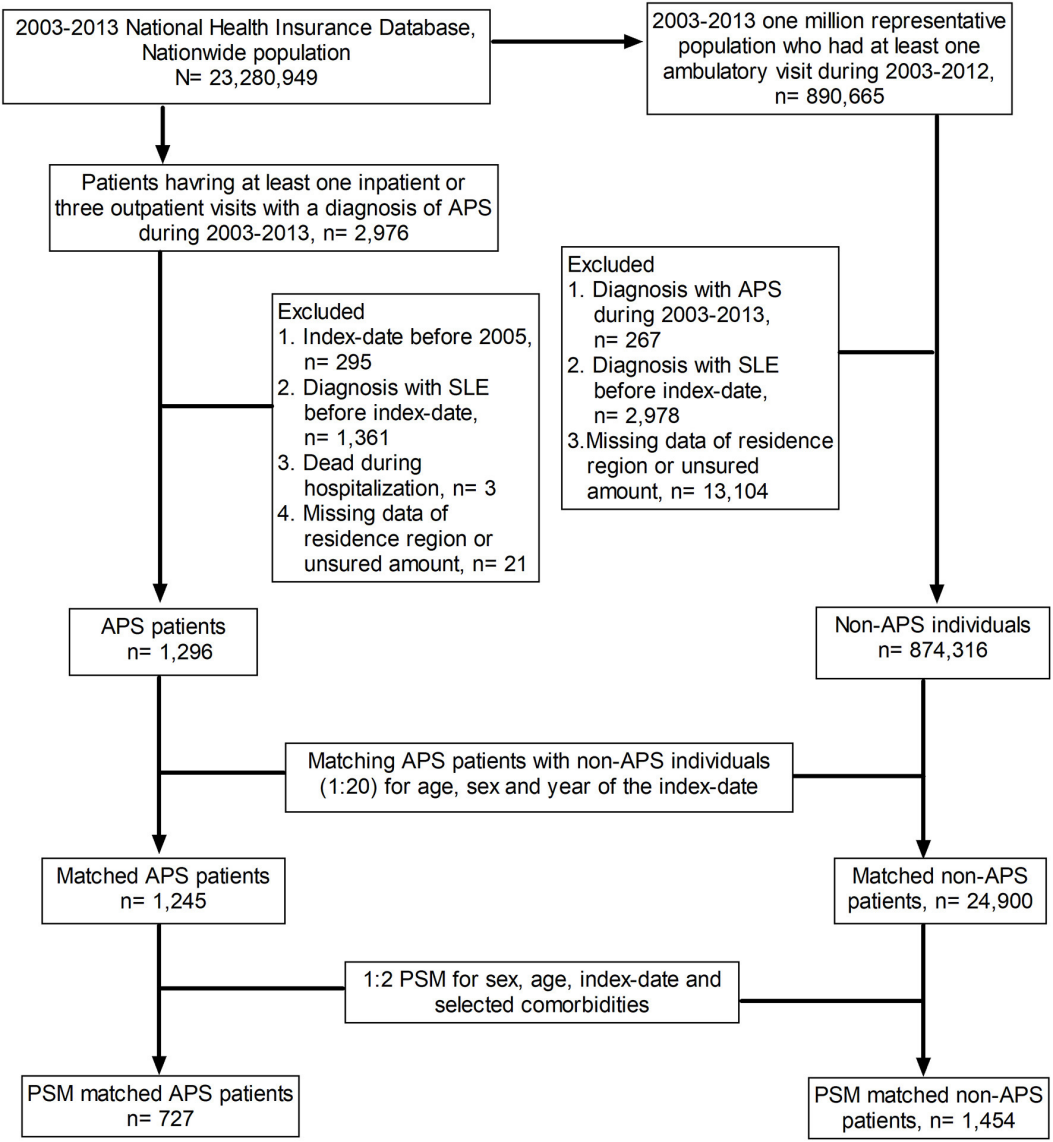
Flow chart of the study design. APS, anti-phospholipid syndrome; SLE, systemic lupus erythematosus; PSM, propensity score matching.

This retrospective study was designed to address the association between anti-phospholipid syndrome (APS) and incident systemic lupus erythematosus (SLE), and the flow-chart is illustrated in Figure 1. The study population was a nationwide population from the period between 2003 and 2013 (*n* = 23,280,949), and we identified patients who had one inpatient or three outpatient visits with a diagnosis of APS between 2003 and 2012 as APS cases (*n* = 2,976). The index date was defined as the date on which APS was first diagnosed in the hospital. The exclusion criteria included the following: (1) index date before 2005 (*n* = 295); (2) diagnosis of SLE before the index date (*n* = 1,361); (3) death during hospitalisation (*n* = 3); and (4) missing data regarding region of residence or insured amount (*n* = 21). A total of 1,296 APS patients met the aforementioned inclusion criteria and were defined as the APS cases in this study. As for the control group, we enrolled individuals who had at least one ambulatory visit during 2005–2012 and applied the following exclusion criteria. (1) an APS diagnosis from 2003 to 2013 (*n* = 267), (2) an SLE diagnosis before the index date (*n* = 2,978), and (3) missing data about region of residence or insured amount (*n* = 13,104). A total of 874,316 individuals were eligible for analyses as the control group. We used age-sex matching and propensity matching in this study. We matched the APS group and the control group at a ratio of 1:20 for sex, age, and year of the index date. After matching, there were 1,245 patients in the APS group and 24,900 individuals in the non-APS group. Moreover, we aimed to reduce the impact of bias and confounding variables on the incidence of SLE through propensity-score matching (PSM), which was conducted at a ratio of 1:2 for sex, age, index date, and selected comorbidities. In the propensity-matched subjects, we identified 727 APS patients and 1,454 control individuals without APS.

### Outcome and Relevant Variables

The ICD-9-CM code of 710.0 was used to identify SLE patients, and ICD-9-CM code of 289.8 was applied for APS. The main outcome of this study was a diagnosis of the above code and at least one hospitalisation or three outpatient visits in a year. We considered baseline comorbidities associated with the risk of developing SLE, including human immunodeficiency virus infection (ICD-9-CM codes 042–044, V08), hyperthyroidism (ICD-9-CM code 242), thyroiditis (ICD-9-CM code 245), diabetes mellitus (ICD-9-CM code 250), hyperlipidemia (ICD-9-CM codes 272.0–272.4), affective psychosis (ICD-9-CM code 296), hypertension (ICD-9-CM codes 401–405), coronary artery disease (ICD-9-CM codes 410–414), vasculitis (ICD-9-CM code 443.0), cerebral vascular accident (ICD-9-CM codes 430–438), chronic obstructive pulmonary disease (ICD-9-CM codes 490–496), asthma (ICD-9-CM code 493), inflammatory bowel disease (ICD-9-CM codes 555–556), pancreatitis (ICD-9-CM codes 577.0 and 577.1), chronic liver diseases (ICD-9-CM codes 571 and 573), chronic kidney disease (ICD-9-CM code 585), rheumatoid arthritis (ICD-9-CM code 714.0), systemic sclerosis (ICD-9-CM code 710.1), Sjogren's syndrome (ICD-9-CM code 710.2), ankylosing spondylitis (ICD-9-CM code 720.0), and osteoporosis (ICD-9-CM code 733). Comorbidities were identified as diseases diagnosed within 1 year before the index date.

### Statistical Analysis

Following matching, we assessed the balance of baseline characteristics in the populations using the absolute standardised difference (ASD). A high degree of balance was reflected by an ASD < 0.1. We counted follow-up person-months and the number of persons diagnosed with SLE, calculated the incidence of SLE (per 100,000 person-months), and estimated the crude relative risk with its 95% confidence interval using Poisson regression. Multivariate Cox proportional hazard regression analysis was then used to estimate the adjusted HR (aHR) for SLE. Four different models were used to investigate the effects of APS exposure and covariates on aHR of SLE, including APS exposure alone, demographic variables, medical utilisation and comorbidities, and a conditional Cox model with APS exposure alone was performed in propensity score-matched populations. Sensitivity analysis was used to estimate the risk of SLE in APS exposure patients who were in age-matched and sex-matched populations under different SLE definitions. Kaplan-Meier curves were generated on the cumulative incidence of SLE in the APS and non-APS groups. The differences between the curves were evaluated using the Log-rank test. In all our studies, *p* < 0.05 was considered statistically significant. All statistical analyses were performed using the Statistical Analysis Software Version 9.4 (SAS Institute Inc., NC, USA).

## Results

### Characteristics of the Study Population

We used the 2003–2013 Taiwanese National Health Insurance Database (NHIRD) to identify 1,296 patients with newly diagnosed APS between 2005 and 2012. We then selected age- and sex-matched (1:20) non-APS subjects, and 1,245 patients with APS as well as 24,900 non-APS controls were eligible for analyses. Furthermore, we selected a comparison group through propensity-score matching (PSM) (1:2) for age, sex, comorbidities, and potential confounders including ITP and AIHA. In the propensity-matched subjects, we enrolled 727 APS patients and 1,454 PSM-matched non-APS controls to address the risk for SLE in patients with APS (Figure 1). Table 1 summaries the baseline characteristics of enrolled subjects with APS and control individuals selected by age-sex matching (1:20) as well as propensity score matching (1:2) (See Supplementary Dataset for details).

**Table 1:** Baseline characteristics in the APS and non-APS groups.

	**Before PSM (1:20 age–sex matching)**			**1:2 PSM**		
	**Non-APS**	**APS**	**ASD**	**Non-APS**	**APS**	**ASD**
	***n* = 24,900**	***n* = 1,245**		***n* = 1,454**	***n* = 727**	
**Sex**			0.000			0.000
Female	19,400 (77.9)	970 (77.9)		1,094 (75.2)	547 (75.2)	
Male	5,500 (22.1)	275 (22.1)		360 (24.8)	180 (24.8)	
**Age (years)**			0.000			0.079
<30	3,960 (15.9)	198 (15.9)		262 (18.0)	140 (19.3)	
30–45	11,680 (46.9)	584 (46.9)		570 (39.2)	299 (41.1)	
45–65	6,720 (27.0)	336 (27.0)		438 (30.1)	216 (29.7)	
≥65	2,540 (10.2)	127 (10.2)		184 (12.7)	72 (9.9)	
**Urbanisation**			0.204			0.070
Urban	8,036 (32.3)	531 (42.7)		647 (44.5)	303 (41.7)	
Suburban	12,041 (48.4)	524 (42.1)		626 (43.1)	319 (43.9)	
Rural	4,823 (19.4)	190 (15.3)		181 (12.4)	105 (14.4)	
Low income^#^	15,463 (62.1)	741 (59.5)	0.053	935 (64.3)	466 (64.1)	0.004
Length of hospital stay^*^			0.598			0.054
0 day	21,448 (86.1)	774 (62.2)		983 (67.6)	503 (69.2)	
1–6 days	2,191 (8.8)	197 (15.8)		210 (14.4)	112 (15.4)	
≥7 days	1,261 (5.1)	274 (22.0)		261 (18)	112 (15.4)	
**Comorbidities^†^**						
No comorbidities	19,314 (77.6)	373 (30.0)	<0.001	775 (53.3)	373 (51.3)	0.131
Comorbidities 1–2	4,190 (16.8)	585 (47)		470 (32.3)	264 (36.3)	
Comorbidities ≥3	1,396 (5.6)	287 (23.1)		209 (14.4)	90 (12.4)	
Rheumatoid arthritis	86 (0.3)	82 (6.6)	0.346	54 (3.7)	32 (4.4)	0.035
Sjogren's syndrome	56 (0.2)	236 (19.0)	0.671	6 (0.4)	10 (1.4)	0.102
Systemic sclerosis	0 (0.0)	18 (1.4)	NA	0 (0.0)	0 (0.0)	
Vasculitis	5 (0.02)	25 (2.0)	0.199	4 (0.3)	2 (0.3)	0.000
Hypertension	2,973 (11.9)	205 (16.5)	0.130	266 (18.3)	118 (16.2)	0.055
Diabetes mellitus	1,431 (5.7)	82 (6.6)	0.035	103 (7.1)	45 (6.2)	0.036
Hyperlipidemia	1,429 (5.7)	136 (10.9)	0.188	191 (13.1)	85 (11.7)	0.044
Thromboembolism	1,129 (4.5)	234 (18.8)		207 (14.2)	97 (13.3)	
Coronary artery disease	717 (2.9)	76 (6.1)	0.156	89 (6.1)	45 (6.2)	0.003
Cerebral vascular accident	467 (1.9)	112 (9.0)	0.318	148 (10.2)	52 (7.2)	0.108
Pulmonary embolism	5 (0.02)	34 (2.7)	0.234	0 (0)	1 (0.1)	NA
Venous thromboembolism	19 (0.1)	45 (3.6)	0.265	6 (0.4)	5 (0.7)	0.037
Portal vein thrombosis	1 (0.004)	4 (0.3)	0.079	1 (0.1)	0 (0)	NA
Arterial embolism and thrombosis	25 (0.1)	16 (1.3)	0.143	6 (0.4)	3 (0.4)	0.000
Pregnancy morbidity	40 (0.2)	90 (7.2)		19 (1.3)	5 (0.7)	
Spontaneous abortion	3 (0.01)	11 (0.9)	0.131	0 (0.0)	0 (0.0)	
Habitual abortion	9 (0.04)	71 (5.7)	0.344	0 (0.0)	0 (0.0)	
Pre-eclampsia/eclampsia	23 (0.1)	11 (0.9)	0.114	15 (1.0)	4 (0.6)	0.054
Abortion	5 (0.02)	4 (0.3)	0.073	4 (0.3)	1 (0.1)	0.030
Infertility	155 (0.6)	178 (14.3)	0.539	93 (6.4)	51 (7.0)	0.025
Raynaud's syndrome	5 (0.02)	25 (2.0)	0.199	4 (0.3)	2 (0.3)	0.000
Thromboangiitis obliterans	2 (0.01)	6 (0.5)	0.096	0 (0.0)	1 (0.1)	NA
Osteoporosis	299 (1.2)	57 (4.6)	0.203	34 (2.3)	21 (2.9)	0.034
Asthma	438 (1.8)	44 (3.5)	0.111	50 (3.4)	25 (3.4)	0.000
Chronic obstructive pulmonary disease	968 (3.9)	90 (7.2)	0.146	109 (7.5)	49 (6.7)	0.029
Chronic kidney disease	147 (0.6)	18 (1.4)	0.085	25 (1.7)	12 (1.7)	0.005
Chronic liver diseases	812 (3.3)	87 (7.0)	0.170	82 (5.6)	44 (6.1)	0.018
Hyperthyroidism	215 (0.9)	35 (2.8)	0.145	29 (2.0)	18 (2.5)	0.033
Thyroiditis	35 (0.1)	80 (6.4)	0.358	17 (1.2)	13 (1.8)	0.051
Pancreatitis	32 (0.1)	18 (1.4)	0.149	19 (1.3)	8 (1.1)	0.019
Affective psychosis	181 (0.7)	38 (3.1)	0.171	36 (2.5)	16 (2.2)	0.018
Ankylosing spondylitis	22 (0.1)	46 (3.7)	0.267	2 (0.1)	4 (0.6)	0.071
Inflammatory bowel disease	58 (0.2)	7 (0.6)	0.052	9 (0.6)	4 (0.6)	0.009
Human immunodeficiency virus	13 (0.1)	0 (0.0)	NA	0 (0.0)	0 (0.0)	
Autoimmune hemolytic anaemia	2 (0.01)	24 (1.9)	0.197	0 (0.0)	0 (0.0)	
Immune thrombocytopenia	1 (0.004)	35 (2.8)	0.240	0 (0.0)	0 (0.0)	
**APS treatment at baseline**						
No drug administration		165 (13.3)			143 (19.7)	
Antiplatelet/anticoagulant only		91 (7.3)			55 (7.6)	
Hydroxychloroquine/corticosteroid +/– Antiplatelet/anticoagulant		989 (79.4)			529 (72.8)	

# *Insured income lower than median income (21,900 New Taiwan dollars)*.

* *Length of hospital stay is defined by hospitalisation days within 2 years of the index date*.

† *Comorbidities are comorbidities identified within 2 years before the index date*.

*APS treatment is treatment received within 6 months after diagnosis with APS*.

*PSM, propensity score-matching; APS, anti-phospholipid syndrome; ASD, absolute standardised difference*.

### Comparison of the Risk for SLE in Subjects With and Without APS

**Figure 2:**
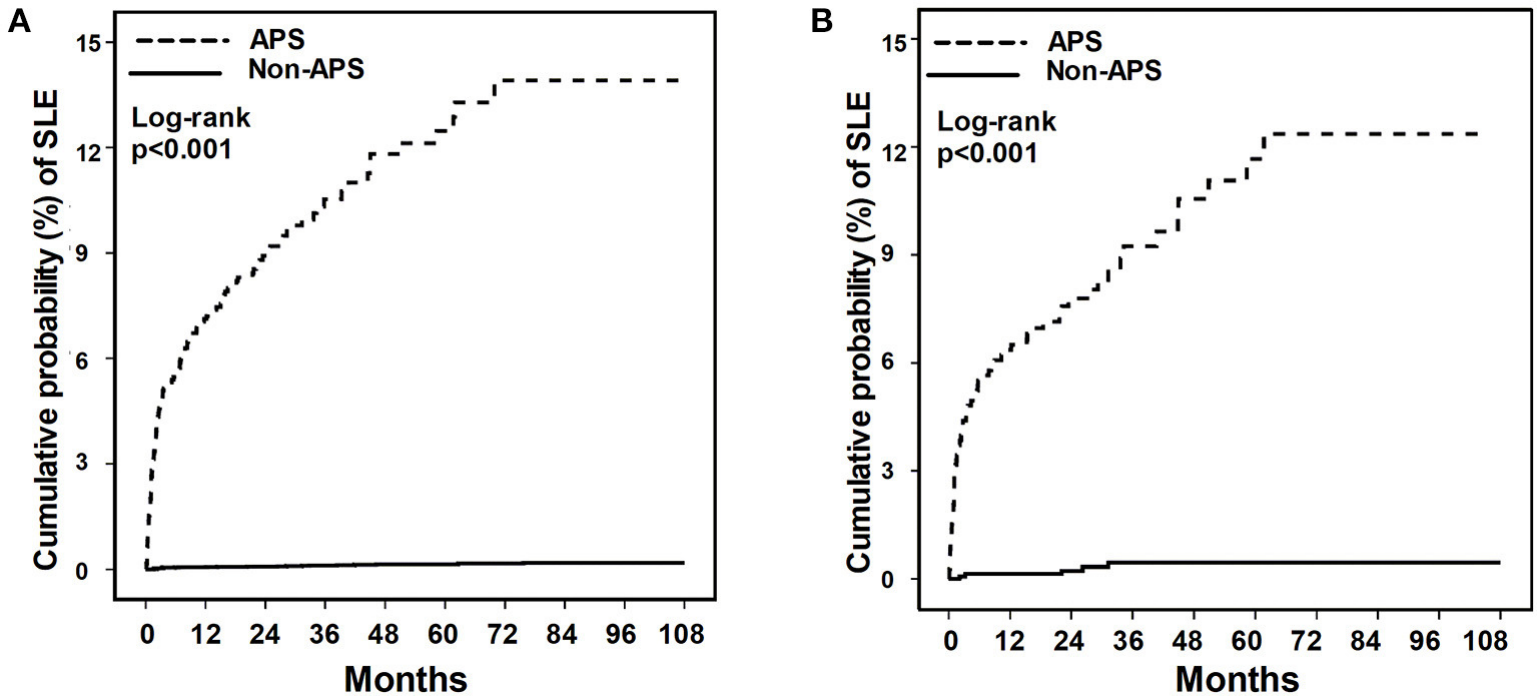
The cumulative incidence of SLE for patients with and without APS. **(A)** age- and sex-matched population. **(B)** Propensity score-matched population. APS, anti-phospholipid syndrome; SLE, systemic lupus erythematosus.

We compared the incidence rate of SLE in patients with and without APS. In the age- and sex-matched subjects, we found a higher incidence rate of SLE (289.79 per 100,000 person-months) in patients with APS compared with the incidence rate of SLE (2.742 per 100,000 person-months) in patients without APS. Similar distinct incident SLE between APS and non-APS controls was found in the propensity-matched subjects, and the incidence rate of SLE in patients with and without APS was 254.15 per 100,000 person-months and 8.19 per 100,000 person-months, respectively (Table 2). After adjustment of the potential confounders, including comorbidities, urbanisation level, history of thromboembolism, pregnancy morbidities, autoimmune hemolytic anaemia, and immune thrombocytopenia, we found that APS was independently associated with incident SLE (HR 80.70; 95% CI 51.37–126.77) (Table 2, Supplementary Table 1). In the propensity-matched subjects, the association between APS and incident SLE remained robust using the conditional Cox regression model (HR 28.55; 95% CI 11.19–70.91). Furthermore, we used Kaplan-Meier plot to demonstrate the time-course of newly diagnosed SLE in patients with and without APS. We found an increased risk for SLE in patients with APS than those without APS and SLE mainly diagnosed within 5 years after the diagnosis of APS (Figure 2).

**Table 2:** Incidence of SLE in the study groups before and after PSM.

	**Before PSM (1:20 age–sex matching)**		**1:2 PSM**	
	**Non-APS**	**APS**	**Non-APS**	**APS**
*n*	24,900	1,245	1,454	727
Follow-up person-months	1,057,555	43,480	61,051	25,576
SLE	29	126	5	65
Incidence rate^*^ (95% CI)	2.742 (2.739–2.745)	289.79 (289.63–289.95)	8.19 (8.17–8.21)	254.15 (253.95–254.34)
Crude relative risk (95% CI)	Reference	105.68 (70.58–158.24)	Reference	31.03 (12.50–77.06)
Adjusted hazard ratio (95% CI)	Reference	80.70 (51.37–126.77)^†^	Reference	28.55 (11.49–70.91)^‡^

* *Incidence rate, per 10,000 person-months*.

*SLE, systemic lupus erythematosus; PSM, propensity score-matching; APS, anti-phospholipid syndrome; CI, confidence interval*.

^†,‡^*Definition of length of hospital day and comorbidities window*.

### Sensitivity Analyses

We performed sensitivity analyses through using distinct definitions of SLE based on SLE treatment and excluding patients who might have secondary APS from non-APS controls (Table 3). We defined SLE by stringent criteria by management with systemic corticosteroid and disease-modifying antirheumatic drugs (DMARDs), and adjHR was 81.12 (95% CI 51.27–128.37) in incident SLE underwent systemic corticosteroid and 82.46 (95% CI 48.82–139.28) in incident SLE underwent DMARDs. The strength of association was consistent with the association between APS and incident SLE without stringent criteria by management (adjHR 80.70, 95% CI 51.37–126.77). Moreover, to mitigate the potential confounding effect of secondary APS on the association between APS and SLE, we hence excluded patients with autoimmune diseases, inflammatory bowel disease, autoimmune hemolytic anaemia and idiopathic thrombocytopenia from the non-APS controls. We found that the association between APS and incident SLE remained consistent after exclusion patient who might has secondary APS (adjHR 88.90; 95% CI 55.21–143.12). Collectively, using a population-based database and propensity matching, we found that patients with APS had a significantly higher risk for SLE compared with those in non-APS controls.

**Table 3:** Sensitivity analysis in the estimation of the SLE risk for APS exposure in age-matched and sex-matched populations.

**Scenario**	**Definition of SLE event**	aHR^*^ (95%CI)
1	At least three outpatient visits or 1 admission within 1 year by rheumatologist (main finding)	80.70 (51.37–126.77)
2	Scenario 1+ treated with systemic corticosteroids or DMARDs (including HCQ or azathioprine)	81.12 (51.27–128.37)
3	Scenario 1+ treated with DMARDs (including HCQ or azathioprine)^†^	82.46 (48.82–139.28)
4	Exclusion of patients with rheumatoid arthritis, Sjogren's syndrome, systemic sclerosis, vasculitis, thyroiditis, ankylosing spondylitis, inflammatory bowel disease, human immunodeficiency virus, AIHA, ITP at baseline (excluding secondary APS)	88.90 (55.21–143.12)

* *aHR of AIHA exposure on the risk of SLE, the covariates including age group, sex, urbanisation, low income, length of hospital stay, and comorbidities listed in Table 1*.

† *The treatment of SLE was identified within 6 months after first diagnosis of SLE*.

*SLE, systemic lupus erythematosus; APS, anti-phospholipid syndrome; aHR, adjusted HR; DMARD, disease modifying antirheumatic drugs; HCQ, hydroxychloroquine; AIHA, autoimmune hemolytic anemias; ITP, immune thrombocytopenia*.

## Discussion

In the present nationwide population-based study, we aimed to address the association between APS and incident SLE using age-sex matching and propensity matching. We identified that diagnosis with APS was highly associated with an increased risk for SLE, and the SLE mainly diagnosed within 5 years after the diagnosis of APS. These findings highlight the essential need of vigilance for SLE in patients diagnosed with APS.

APS is highly associated with SLE and may affect the outcome in patients with SLE (10–12). Unlike SLE, which is the prototypical autoimmune disease with a wide range of anti-nuclear antibodies and clinical presentations, APS mainly manifested with thrombotic events and a positive anti-phospholipid antibody (13). Therefore, there is a crucial need to explore the risk of incident SLE in patients with APS. In line with our findings, Freire et al., using an APS cohort with 80 APS patients, reported that 17.5% (14/80) of patients with primary APS evolved into SLE within 5.2 ± 4 years (14). Given that thrombotic event mainly managed by non-rheumatologists, the aforementioned evidence highlight the need for collaboration with the rheumatologist for subtle signs of SLE, particularly in the first few years after the diagnosis of APS.

Anti-phospholipid syndrome (APS) is characterised by vascular thrombosis, including venous and arterial thrombosis (6). Intriguingly, Zuily et al. recently conducted a hierarchical cluster analysis using 30 data points among 497 anti-phospholipid antibody-positive patients in the Alliance for Clinical Trials and International Networking (APS ACTION) registry and reported three main phenotypes, including female patients without autoimmune diseases but with venous thrombosis (36.0%, 179/497), female patients with SLE, thrombocytopaenia and haemolytic anaemia (36.2%, 180/497), and older men with arterial thrombosis and cardiovascular manifestations (27.8%, 138/497) (15). The aforementioned finding highlights the high correlation between SLE and hametological abnormalitie as shown in recent studies including our previous study and the present study (1, 2), and we further specified the risk and time-course of incident SLE in APS patients. Additionally, in line with the finding of Zuily et al. we also found that venous thromboembolism (adjHR 2.67, 95% CI 1.34–5.32) and portal vein thrombosis (adjHR 4.78, 95% CI 1.06–21.53), instead of arterial and cardiovascular thrombosis, was independently associated with incident SLE in patients with APS (Supplementary Table 1).

Indeed, mechanisms underlie evolution into over SLE in patients with APS remain a research niche. The development of SLE involves a gradual loss of tolerance to self-antigens, followed by an autoantibodies production (16). Genetic susceptibility and environmental exposure, have been implicated with the development of SLE (17–20). Distinct Human leukocyte antigen-DRB1 and -DQB1 allele was reported to be associated with APS with and without SLE, and more studies are required to elucidate the genetic basis of APS-SLE (17, 21, 22). A number of environmental factors, including diet, medication, pollutant, vaccination and microbial infection, and complex gene-environment interaction have been implicated with the development of autoimmunity (20). Currently, hypercoagulation has been increasingly reported in patients with coronavirus COVID-19 infection (23). Given that COVID-19 infection and SLE are both implicated with dysregulated immune responses, such as type I interferon pathway, there are increasing studies to address the correlation among autoimmune disease, hypercoagulation and COVID-19 infection (24–27). These evidence highlight the substantial needs for studies to clarify the underlying biological mechanism for the development of SLE in patients with APS.

SLE is associated with ITP and APS; however, it is somehow difficult to delineate APS and ITP among patients with thrombocytopenia (28). In one population-based study aiming to address the association between ITP and SLE, Zhu et al. reported that patients with ITP were more likely to have APS compared with those without ITP (2.77 vs. 0.02%, *p* < 0.05) (1). Thrombocytopenia is a cardinal haematological manifestation of APS, but the presence of thrombocytopenia, the main manifestation in patients with ITP, does not exclude the risk for the development of thrombosis (28). In the present study, we found a consistent association between APS and incident SLE using regression adjusted with ITP diagnosis and excluded patients with ITP. Therefore, APS should be an independent risk factor for incident SLE.

There are limitations in this study. First, the lack of laboratory data to validate the diagnosis of SLE in the claim database. However, we have conducted sensitivity analyses and used stringent criteria with concomitant management with systemic corticosteroid and DMARDs, and the strength of association between APS and incident is consistent. Therefore, the concern regarding the diagnosis of SLE should be at least partly mitigated. Second, the diagnosis of APS without laboratory data should also be a concern. Given that APS is relatively a specific diagnosis in patients with thromboembolism, we think that the diagnosis of APS with one inpatient or three outpatient visits could mainly be underestimated, instead of overestimated, in the present study. The strength of the study was the use of nationwide, population-based database to minimise the risk of selection and participation bias although clinical features and laboratory data, including anti-nuclear antibody and APS profiles, are not available in NHIRD. Third, the undiagnosed SLE in the diagnosis of APS could also be a concern, and we believe this concern somehow indicates the needs for collaboration with the rheumatologist in the management of patients with APS. Fourth, the finding of the present study should be further validated in another independent cohort.

## Conclusion

In conclusion, using a population-based study, we demonstrate a high risk of developing SLE among APS patients, in particular during the first 5 years after APS diagnosis. These findings highlight the substantial need for close monitoring for SLE among patients with APS. More studies are warranted to explore factors including genetic and environmental factors leading to SLE in patients with APS.

## Data Availability Statement

The original contributions presented in the study are included in the article/Supplementary Material, further inquiries can be directed to the corresponding author/s.

## Ethics Statement

This study was approved by the Institutional Review Board of Taichung Veterans General Hospital in Taiwan (approval number: CE17100B). Written informed consent for participation was not required for this study in accordance with the national legislation and the institutional requirements.

## Author Contributions

H-HC and W-CC conceptualised the research and drafted the manuscripts. H-HC, C-HL, and W-CC acquired and interpreted the data. All authors have read and approved the final manuscript.

## Conflict of Interest

The authors declare that the research was conducted in the absence of any commercial or financial relationships that could be construed as a potential conflict of interest.

## References

[1] Zhu FX Huang JY Ye Z Wen QQ Wei JC. Risk of systemic lupus erythematosus in patients with idiopathic thrombocytopenic purpura: a population-based cohort study. Ann Rheum Dis. (2020) 79:793–99. 10.1136/annrheumdis-2020-217013 32241798

[2] Mo HY Wei JCC Chen XH Chen HH. Increased risk of systemic lupus erythematosus in patients with autoimmune haemolytic anaemia: a nationwide population-based cohort study. Ann Rheum Dis. (2020) 80:403–4. 10.1136/annrheumdis-2020-219328 32963051

[3] Tincani A Andreoli L Chighizola C Meroni PL. The interplay between the antiphospholipid syndrome and systemic lupus erythematosus. Autoimmunity. (2009) 42:257–9. 10.1080/08916930902827918 19811269

[4] Belizna C Stojanovich L Cohen-Tervaert JW Fassot C Henrion D Loufrani L. Primary antiphospholipid syndrome and antiphospholipid syndrome associated to systemic lupus: are they different entities? Autoimmun Rev. (2018) 17:739–45. 10.1016/j.autrev.2018.01.027 29885541

[5] Shoenfeld Y Meroni PL Toubi E. Antiphospholipid syndrome and systemic lupus erythematosus: are they separate entities or just clinical presentations on the same scale? Curr Opin Rheumatol. (2009) 21:495–500. 10.1097/BOR.0b013e32832effdd 19593144

[6] Giannakopoulos B Krilis SA. The pathogenesis of the antiphospholipid syndrome. N Engl J Med. (2013) 368:1033–44. 10.1056/NEJMra1112830 23484830

[7] Cervera R Serrano R Pons-Estel GJ Ceberio-Hualde L Shoenfeld Y de Ramon E. Morbidity and mortality in the antiphospholipid syndrome during a 10-year period: a multicentre prospective study of 1000 patients. Ann Rheum Dis. (2015) 74:1011–8. 10.1136/annrheumdis-2013-204838 24464962

[8] Tarr T Lakos G Bhattoa HP Szegedi G Shoenfeld Y Kiss E. Primary antiphospholipid syndrome as the forerunner of systemic lupus erythematosus. Lupus. (2007) 16:324–8. 10.1177/0961203307077993 17576733

[9] Unlu O Erkan D Barbhaiya M Andrade D Nascimento I Rosa R. The impact of systemic lupus erythematosus on the clinical phenotype of antiphospholipid antibody-positive patients: results from the antiphospholipid syndrome alliance for clinical trials and international clinical database and repository. Arthritis Care Res. (2019) 71:134–41. 10.1002/acr.23584 29669399 PMC6484425

[10] Meroni PL Tsokos GC. Editorial: systemic lupus erythematosus and antiphospholipid syndrome. Front Immunol. (2019) 10:199. 10.3389/fimmu.2019.00199 30858846 PMC6398508

[11] Ruiz-Irastorza G Egurbide MV Ugalde J Aguirre C. High impact of antiphospholipid syndrome on irreversible organ damage and survival of patients with systemic lupus erythematosus. Arch Intern Med. (2004) 164:77–82. 10.1001/archinte.164.1.77 14718326

[12] Torricelli AK Ugolini-Lopes MR Bonfa E Andrade D. Antiphospholipid syndrome damage index (DIAPS): distinct long-term kinetic in primary antiphospholipid syndrome and antiphospholipid syndrome related to systemic lupus erythematosus. Lupus. (2020) 29:256–62. 10.1177/0961203320901598 31986962

[13] Agmon-Levin N Shoenfeld Y. The spectrum between antiphospholipid syndrome and systemic lupus erythematosus. Clin Rheumatol. (2014) 33:293–5. 10.1007/s10067-014-2486-5 24435353

[14] Freire PV Watanabe E dos Santos NR Bueno C Bonfa E de Carvalho JF. Distinct antibody profile: a clue to primary antiphospholipid syndrome evolving into systemic lupus erythematosus? Clin Rheumatol. (2014) 33:349–53. 10.1007/s10067-013-2472-3 24420722

[15] Zuily S Clerc-Urmes I Bauman C Andrade D Sciascia S Pengo V. Cluster analysis for the identification of clinical phenotypes among antiphospholipid antibody-positive patients from the APS ACTION registry. Lupus. (2020) 29:961203320940776. 10.1177/0961203320940776 32703117 PMC8216235

[16] Leffers HCB Lange T Collins C Ulff-Moller CJ Jacobsen S. The study of interactions between genome and exposome in the development of systemic lupus erythematosus. Autoimmun Rev. (2019) 18:382–92. 10.1016/j.autrev.2018.11.005 30772495

[17] Rullo OJ Tsao BP. Recent insights into the genetic basis of systemic lupus erythematosus. Ann Rheum Dis. (2013) 72 (Suppl. 2):ii56–61. 10.1136/annrheumdis-2012-202351 23253915 PMC3780983

[18] Armstrong DL Zidovetzki R Alarcon-Riquelme ME Tsao BP Criswell LA Kimberly RP. GWAS identifies novel SLE susceptibility genes and explains the association of the HLA region. Genes Immun. (2014) 15:347–54. 10.1038/gene.2014.23 24871463 PMC4156543

[19] Ceccarelli F Perricone C Borgiani P Ciccacci C Rufini S Cipriano E. Genetic factors in systemic lupus erythematosus: contribution to disease phenotype. J Immunol Res. (2015) 2015:745647. 10.1155/2015/745647 26798662 PMC4699011

[20] Kamen DL. Environmental influences on systemic lupus erythematosus expression. Rheum Dis Clin North Am. (2014) 40:401–12, vii. 10.1016/j.rdc.2014.05.003 25034153 PMC4198387

[21] Kapitany A Tarr T Gyetvai A Szodoray P Tumpek J Poor G. Human leukocyte antigen-DRB1 and -DQB1 genotyping in lupus patients with and without antiphospholipid syndrome. Ann N Y Acad Sci. (2009) 1173:545–51. 10.1111/j.1749-6632.2009.04642.x 19758197

[22] Ortiz-Fernandez L Sawalha AH. Genetics of antiphospholipid syndrome. Curr Rheumatol Rep. (2019) 21:65. 10.1007/s11926-019-0869-y 31807905

[23] Helms J Tacquard C Severac F Leonard-Lorant I Ohana M Delabranche X. High risk of thrombosis in patients with severe SARS-CoV-2 infection: a multicenter prospective cohort study. Intensive Care Med. (2020) 46:1089–98. 10.1007/s00134-020-06062-x 32367170 PMC7197634

[24] Novelli L Motta F De Santis M Ansari AA Gershwin ME Selmi C. The JANUS of chronic inflammatory and autoimmune diseases onset during COVID-19 - a systematic review of the literature. J Autoimmun. (2020) 117:102592. 10.1016/j.jaut.2020.102592 33401171 PMC7833462

[25] Xourgia E Tektonidou MG. Type I interferon gene expression in antiphospholipid syndrome: pathogenetic, clinical and therapeutic implications. J Autoimmun. (2019) 104:102311. 10.1016/j.jaut.2019.102311 31378637

[26] Kamel MH Yin W Zavaro C Francis JM Chitalia VC. Hyperthrombotic milieu in COVID-19 patients. Cells. (2020) 9:2392. 10.3390/cells9112392 PMC7694011 33142844

[27] Talarico R Aguilera S Alexander T Amoura Z Antunes AM Arnaud L. The impact of COVID-19 on rare and complex connective tissue diseases: the experience of ERN ReCONNET. Nat Rev Rheumatol. (2021) 17:177–84. 10.1038/s41584-020-00565-z 33408338 PMC7786339

[28] Artim-Esen B Diz-Kucukkaya R Inanc M. The significance and management of thrombocytopenia in antiphospholipid syndrome. Curr Rheumatol Rep. (2015) 17:14. 10.1007/s11926-014-0494-8 25740703

