# Supplementary material for: Risk of Systemic Lupus Erythematosus in Patients With Anti-phospholipid Syndrome: A Population-Based Study
Source: Front Med (Lausanne). 2021 May 10;8:654791. doi: 10.3389/fmed.2021.654791 (PMC8141575; doi:10.3389/fmed.2021.654791)
Supplement: Supplementary file 1 [file Table_1.DOCX]

| **Supplemental table 1. Cox proportional hazard regressions for estimation of adjusted HRs on SLE** | | | | | |
| --- | --- | --- | --- | --- | --- |
|  | **1:20 age-matched and sex-matched population** | | |  | **1:2 PSM population** |
|  | **Model 1: APS exposure alone** | **Model 2: APS exposure + demographic variables** | **Model 3: model 2 + medical utilization and comorbidities at baseline** |  | **Model 4: conditional cox model with APS exposure alone** |
| APS | 96.08 (64.14–143.92) | 99.83 (66.60–149.62) | 80.70 (51.37–126.77) |  | 28.55 (11.49–70.91) |
| Sex: Female |  | 1.03 (0.70–1.52) | 0.87 (0.57–1.32) |  |  |
| Age (years) |  |  |  |  |  |
| <30 |  | Ref. | Ref. |  |  |
| 30–45 |  | 0.96 (0.63–1.48) | 0.96 (0.62–1.50) |  |  |
| 45–65 |  | 0.79 (0.49–1.26) | 0.61 (0.35–1.05) |  |  |
| ≥65 |  | 0.55 (0.28–1.07) | 0.39 (0.18–0.84) |  |  |
| Urbanization |  |  |  |  |  |
| Urban |  | Ref. | Ref. |  |  |
| Suburban |  | 1.66 (1.15–2.39) | 1.60 (1.10–2.33) |  |  |
| Rural |  | 1.14 (0.68–1.89) | 0.90 (0.53–1.53) |  |  |
| Low income |  | 1.76 (1.22–2.56) | 1.82 (1.24–2.66) |  |  |
| Length of hospital stay* |  |  |  |  |  |
| 0 day |  |  | Ref. |  |  |
| 1–6 days |  |  | 1.44 (0.93–2.23) |  |  |
| ≥7 days |  |  | 1.06 (0.66–1.71) |  |  |
| Co-morbidity† |  |  |  |  |  |
| Rheumatoid arthritis |  |  | 0.90 (0.41–1.97) |  |  |
| Sjogren’s syndrome |  |  | 1.11 (0.68–1.81) |  |  |
| Systemic sclerosis |  |  | Cannot estimate |  |  |
| Vasculitis |  |  | 1.44 (0.39–5.31) |  |  |
| Hypertension |  |  | 1.00 (0.57–1.74) |  |  |
| Diabetes mellitus |  |  | 1.25 (0.63–2.48) |  |  |
| Hyperlipidemia |  |  | 1.41 (0.79–2.50) |  |  |
| Thromboembolism |  |  |  |  |  |
| Coronary artery disease |  |  | 0.86 (0.37–1.97) |  |  |
| Cerebral vascular accident |  |  | 1.41 (0.81–2.46) |  |  |
| Pulmonary embolism |  |  | 1.73 (0.76–3.94) |  |  |
| Venous thromboembolism |  |  | 2.67 (1.34–5.32) |  |  |
| Portal vein thrombosis |  |  | 4.78 (1.06–21.53) |  |  |
| Arterial embolism and thrombosis |  |  | 0.66 (0.15–3.02) |  |  |
| Pregnancy morbidity |  |  |  |  |  |
| Spontaneous abortion |  |  | 2.42 (0.58–10.11) |  |  |
| Habitual abortion |  |  | 0.91 (0.39–2.16) |  |  |
| Preeclampsia/eclampsia |  |  | 1.15 (0.15–8.63) |  |  |
| Abortion |  |  | Cannot estimate |  |  |
| Infertility |  |  | 0.84 (0.44–1.61) |  |  |
| Raynaud’s syndrome |  |  | Cannot estimate |  |  |
| Thromboangiitis obliterans |  |  | 4.46 (0.87–22.96) |  |  |
| Osteoporosis |  |  | 1.02 (0.44–2.41) |  |  |
| Asthma |  |  | 1.12 (0.35–3.62) |  |  |
| Chronic obstructive pulmonary disease |  |  | 1.04 (0.43–2.49) |  |  |
| Chronic kidney disease |  |  | 1.00 (0.24–4.22) |  |  |
| Chronic liver diseases |  |  | 1.52 (0.84–2.75) |  |  |
| Hyperthyroidism |  |  | 0.62 (0.15–2.63) |  |  |
| Thyroiditis |  |  | 0.30 (0.07–1.27) |  |  |
| Pancreatitis |  |  | 1.22 (0.29–5.18) |  |  |
| Affective psychosis |  |  | 1.30 (0.55–3.10) |  |  |
| Ankylosing spondylitis |  |  | 0.20 (0.03–1.46) |  |  |
| Inflammatory bowel disease |  |  | Cannot estimate |  |  |
| Human immunodeficiency virus |  |  | Cannot estimate |  |  |
| Autoimmune hemolytic anemia |  |  | 1.84 (0.82–4.15) |  |  |
| Immune thrombocytopenia |  |  | 3.53 (1.79–6.98) |  |  |
| *Length of hospital stay is defined by hospitalization days within 2 years of the index date.  †Comorbidities are comorbidities identified within 2 years before the index date.  ‡APS treatment is treatment received within 6 months after diagnosis with APS.  PSM, propensity score-matching; APS, antiphospholipid syndrome; ASD, absolute standardized difference. | | | | | |
